# Novel Potential Biomarker of Adult Cardiac Surgery-Associated Acute Kidney Injury

**DOI:** 10.3389/fphys.2020.587204

**Published:** 2020-11-10

**Authors:** Zujun Chen, Zhenliang Hu, Yiqing Hu, Yixuan Sheng, Yuan Li, Jiangping Song

**Affiliations:** ^1^State Key Laboratory of Cardiovascular Disease, Department of Cardiac Surgery, National Center for Cardiovascular Diseases, Fuwai Hospital, Chinese Academy of Medical Sciences and Peking Union Medical College, Beijing, China; ^2^Department of Cardiovascular Surgery, Guangzhou First People’s Hospital, School of Medicine, South China University of Technology, Guangzhou, China

**Keywords:** biomarker, acute kidney injury, cytokines, IFN-γ, SCGF-β

## Abstract

**Background:**

Acute kidney injury (AKI) occurs in about 30% of patients with cardiac surgery, but the pathogenesis of cardiac surgery-associated acute kidney injury (CSA-AKI) remains unclear and there are no predictive biomarkers or diagnostic criteria specific for CSA-AKI beyond the general clinical variables for AKI like serum creatinine (SCr).

**Methods and Results:**

We measured the plasma levels of 48 cytokines within 24 h after cardiac surgery in a total of 306 adult patients including 204 with and 102 without AKI, and then evaluated the diagnostic efficacy of these cytokines for the development of CSA-AKI via ANOVA and Pearson correlation analysis. Among these 48 cytokines, 20 of them were significantly different in the AKI patients compared with the non-AKI patients. In particularly, 13 cytokines displayed tremendous changes with the *P* < 1E^–5^. Moreover, 10 of the 48 cytokines in the plasma were significantly different among the patients with different stages of AKI. Specifically, 6 cytokines exhibited immense differences with the *P* < 1E^–5^. Additionally, 7 of the 48 cytokines have the correlation coefficient of *r* > 0.5 with the postoperative changes of SCr after cardiac surgery.

**Conclusion:**

Taken all the results together, IFN-γ and SCGF-β were the most relevant two cytokines that were not only remarkably changed in adult CSA-AKI patients during the first 24 h after cardiac surgery, but also significantly correlated with the postoperative changes of SCr after cardiac surgery. Therefore, IFN-γ and SCGF-β might be novel predictive plasma biomarker, as well as potential therapeutic targets specific for adult CSA-AKI.

## Introduction

Acute renal injury (AKI), characterized by an abrupt loss of kidney function with sudden reduction of glomerular filtration rate (GFR) as well as dramatic retention of nitrogenous waste products, is a common clinical syndrome after cardiac surgery with an incidence of about 30%. The cardiac surgery-associated acute kidney injury (CSA-AKI) is associated with a series of postoperative adverse events, including prolonged intensive care, extended hospital stay, and increased patient mortality (Brinkman et al., 2015). According to the Kidney Disease Improving Global Outcomes (KDIGO), the diagnosis of AKI is generally based on a criteria of an increase in serum creatinine (SCr) by ≥0.3 mg/dl (26.5 μmol/L) within 48 h or an increase in SCr to 1.5 times of the baseline (Arsalan et al., 2018). Even though significant differences exist between CSA-AKI and other types of AKI in etiology, pathophysiology, symptoms and treatment, there is currently no available predictive biomarkers or diagnostic criteria specific for CSA-AKI beyond the general clinical variables for AKI, such as SCr and cystatin C.

A recent prospective cohort study assessed the correlation of a set of urinary and plasma biomarkers with the development of AKI in 408 children from 1 month old to 18 years old undergoing cardiac surgery. They found that among these biomarkers, plasma IL-8, a potent proinflammatory cytokine, exhibited the best discrimination for pediatric CSA-AKI (Greenberg et al., 2018). Several studies have also shown the association of some urinary or plasma biomarkers with the tubular damage or inflammatory response in adult CSA-AKI, but the prediction probability of these factors for the progression of AKI following cardiac surgery in adult patients have not been evaluated yet (Burke-Gaffney et al., 2014; Brinkman et al., 2015; Greenberg et al., 2015; de Fontnouvelle et al., 2017; Arsalan et al., 2018).

In the present study, we measured the plasma levels of 48 cytokines within 24 h after cardiac surgery in 204 adult AKI patients, as well as 102 non-AKI patients, and then evaluated the diagnostic efficacy of these cytokines for the development of CSA-AKI via ANOVA and Pearson correlation analysis.

## Materials and Methods

### Study Design

**FIGURE 1:**
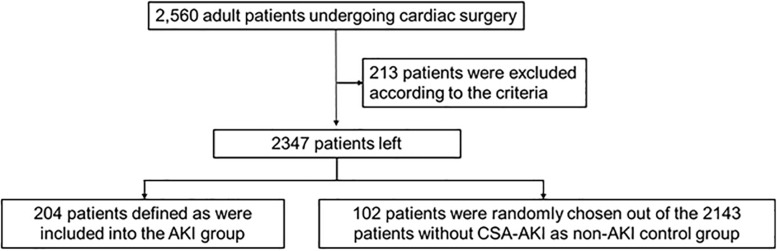
Flowchart of patients selection.

This study was approved by the ethics committee of Fuwai hospital and all participants signed the informed consent. A total of 2,560 adult patients undergoing cardiac surgery at Fuwai hospital were registered during the enrollment period from 12/6/2017 to 11/27/2018, of which 213 patients were excluded according to the criteria including preoperative history of renal insufficiency, preoperative creatinine levels ≥2 times of the age-adjusted normal range, or ≥2 cardiac surgeries within a year. Among the remaining 2,347 patients, 204 patients were defined as CSA-AKI and all of them were included into the AKI group. Then, 102 patients were randomly chosen out of the 2,143 patients without CSA-AKI as non-AKI control group (Figure 1).

Peripheral blood sample was collected within the first 24 h after cardiac surgery and then centrifuged at 8,000 rpm for 5 min at 4°C to separate plasma. The baseline Src was measured within 72 h before surgery. All participants’ demographic characteristics, medical histories, surgical procedures, as well as preoperative and postoperative clinical outcomes were recorded. The primary clinical outcomes were the occurrence and severity of AKI defined by the rise of SCr following cardiac surgery (non-AKI: < 1.5-fold; stage I AKI: 1.5–1.9-fold; stage II AKI: 2–2.9-fold; stage III AKI: ≥ 3-fold) (Mehta et al., 2007). The secondary clinical outcomes included the length of ICU and hospital stay, ventilator use, hemodialysis, renal replacement therapy, as well as hospitalization death.

### Cytokine Array Measurement

The concentrations of a total of 48 cytokines including FGF basic, Eotaxin, G-CSF, GM-CSF, IFN-γ, IL-1β, IL-1Ra, IL-1α, IL-2Rα, IL-3, IL-12 (p40), IL-16, IL-2, IL-4, IL-5, IL-6, IL-7, IL-8, IL-9,GRO-α, HGF, IFN-α2, LIF, MCP-3, IL-10, IL-12 (p70), IL-13, IL-15, IL-17A, IP-10, MCP-1 (MCAF), MIG, β-NGF, SCF, SCGF-β, SDF-1α, MIP-1α, MIP-1β, PDGF-BB, RANTES, TNF-α, VEGF, CTACK, MIF, TRAIL, IL-18, M-CSF, and TNF-β in the plasma samples were measured with a Human Cytokine Screening Panel (12007283, Bio-Rad, United States) according to the manufacturer’s instructions.

### Statistical Analysis

Analysis of variance (ANOVA) was used to identify significantly changed cytokines between CSA-AKI and non-AKI groups. In order to exclude the possibility that variation in sex and age might influence the detection of significant cytokines, we included these two factors as covariates within our ANOVA model. Resulting *P*-values from ANOVA were corrected for multiple comparisons using the Bonferroni correction. A heatmap was plotted to display the concentration of these significantly changed cytokines in different groups. A Pearson correlation coefficient was calculated to evaluate the association between cytokine concentration and ΔSCr. We use regression analysis to calculate the prediction probability of factors combination, which was used to draw the ROC (receiver-operating characteristic curve) curve consequently to display the performance. Pearson correlation analysis was used to determine the relationship between the biomarkers and the clinical events. All statistical analysis was performed in R.

## Results

### The Most Significantly Changed Plasma Cytokines in the CSA-AKI Patients

**FIGURE 2:**
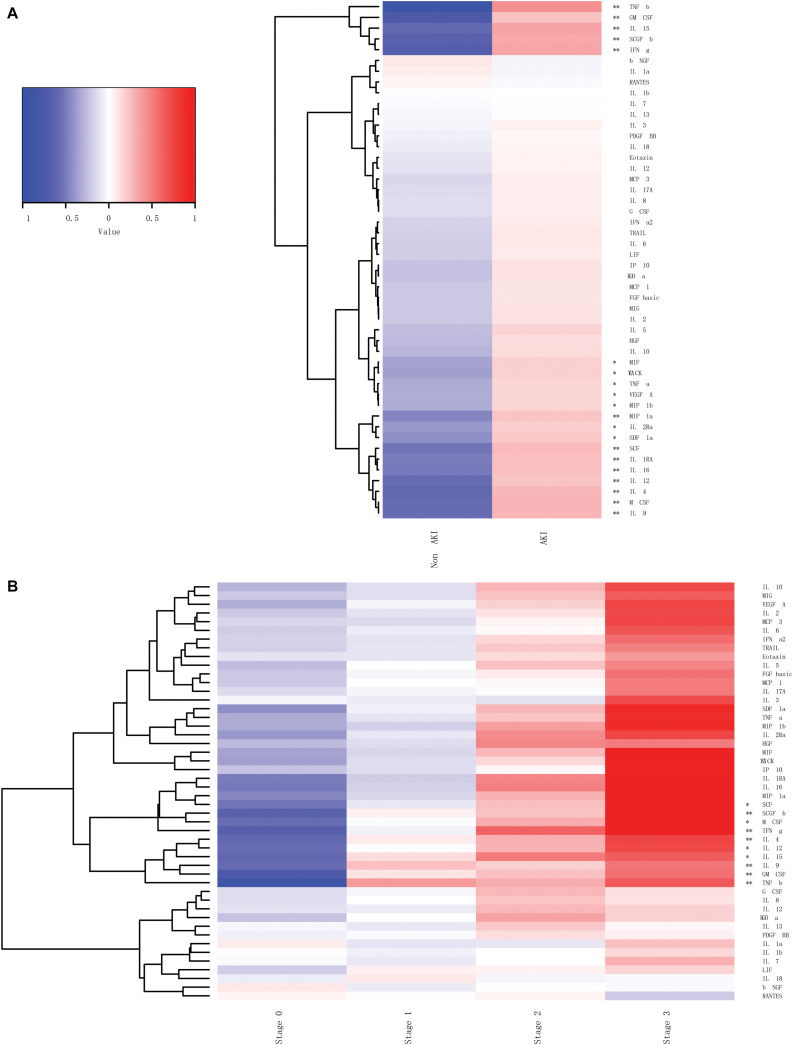
The plasma concentrations of 48 cytokines within 24 h after cardiac surgery. **(A)** The plasma concentrations of 20 cytokines were significantly different between the AKI and non-AKI groups. **(B)** The plasma concentrations of these 48 cytokines between the groups with different AKI stages. *p < 0.05, **p < 0.00001.

We measured the plasma concentrations of 48 cytokines within 24 h after cardiac surgery in 204 adult patients with CSAAKI and 102 patients without CSA-AKI (Table 1). We found that the plasma concentrations of 20 cytokines were significantly different between the AKI and non-AKI groups with the *P* < 0.05, in which 13 cytokines including TNF-β, IFN-γ, SCGF-β, IL-15, IL-9, IL-4, M-CSF, GM-CSF, SCF, IL-16, IL-12, IL-1RA, and MIP-1α, were the most significantly changed cytokines with the *P* < 1E^–5^ (Figures 2A, 4, Supplementary Figure S1, and Supplementary Table S1).

**TABLE 1:** Baseline characteristics of patients with non-AKI and AKI (*N* = 306).

Characteristics	Non-AKI (*n* = 102)	AKI (*N* = 204)	*P*
Male, n (%)	88 (86.27%)	149 (73.04%)	0.0090
Age (years)	58.91 ± 1.00	55.90 ± 0.88	0.0598
Height (cm)	169.80 ± 0.69	169.76 ± 0.59	0.6663
Weight (kg)	73.70 ± 1.10	75.07 ± 1.10	0.9759
Body weight index (BMI) (kg/m^2^)	25.53 ± 0.32	25.97 ± 0.32	0.7360
Diabetes (n)	23 (22.55%)	27 (13.24%)	0.0485
Hyperlipidemia (n)	61 (59.80%)	103 (50.73%)	0.1459
Hypertension (n)	65 (63.73%)	135 (66.18%)	0.7031
Cardiopulmonary Bypass, n (%)	73 (71.57%)	170 (83.33%)	0.0239
Bypass time (minute)	112.05 ± 4.79	157.72 ± 6.52	<0.0001
Aortic cross clamp time (minute), (n)	81.12 ± 3.69	99.17 ± 3.89 (123)	0.0027
Preoperative Src (μmol/L)	88.78 ± 2.08	89.54 ± 1.35	0.4419
LVEF (%)	58.99 ± 0.74	60.57 ± 0.42	0.1880
NYHA			0.0059
I (%)	31 (30.39%)	86 (42.16%)	
II (%)	51 (50.00%)	61 (29.90%)	
III (%)	20 (19.61%)	55 (26.96%)	
IV (%)	0	2 (0.98%)	
ICU LOS (d)	3.43 ± 0.34	5.94 ± 0.40	<0.0001
Hospitalization	15.37 ± 0.80	19.46 ± 0.80	0.0013
Ventilator time (h)	19.83 ± 1.63	55.60 ± 5.51	<0.0001
Dialysis (n)	1	26	0.0002
In-hospital death or therapy failure (n)	1	15	0.0074

*Data are expressed as either Mean ± SDE or as number and percentage. ICU LOS, Length of ICU stay; LVEF, left ventricular ejection fraction.*

We also compared the plasma concentrations of These 48 cytokines between the non-AKI groups with different AKI stages (Table 2). We found that the plasma concentrations of 10 cytokines were significantly different among the non-AKI, Stage I AKI, Stage II AKI, and Stage III AKI groups, in which 6 cytokines including TNF-β, SCGF-β, IL-9, IFN-γ, GM-CSF, and IL-4, were the most significantly changed cytokines with the *P* < 1E^–5^ (Figures 2B, 5, Supplementary Figure S2, and Supplementary Table S1).

**TABLE 2:** Baseline characteristics of patients with non-AKI and stage I, II, III AKI, respectively (*N* = 306).

Characteristics	Non-AKI (*n* = 102)	Stage I AKI (*N* = 115)	Stage II AKI (*N* = 49)	Stage III AKI (*N* = 40)	*P*
Male, n (%)	88 (86.27%)	95 (82.60%)	29 (59.18%)	25 (62.50%)	0.0001
Age (years)	58.91 ± 1.00	56.70 ± 1.15	56.88 ± 1.58	52.38 ± 2.23	0.0806
Height (cm)	169.80 ± 0.69	170.03 ± 0.73	168.04 ± 1.33	171.08 ± 1.40	0.5154
Weight (kg)	73.70 ± 1.10	75.21 ± 1.21	74.26 ± 2.92	75.67 ± 2.73	0.5456
Body weight index (BMI) (kg/m^2^)	25.53 ± 0.32	25.96 ± 0.34	26.19 ± 0.87	25.73 ± 0.76	0.8522
Diabetes (n)	23 (22.55%)	20 (17.39%)	7 (14.29%)	0	0.0097
Hyperlipidemia (n)	61 (59.80%)	58 (50.43%)	29 (59.18%)	16 (40.00%)	0.1310
Hypertension (n)	65 (63.73%)	73 (63.49%)	36 (73.47%)	26 (65.00%)	0.6312
Cardiopulmonary Bypass, n (%)	73 (71.57%)	100 (84.03%)	41 (83.67%)	29 (72.50%)	0.0228
Bypass time (minute)	112.05 ± 4.79	144.95 ± 6.16^c^	165.96 ± 17.27^b^	187.48 ± 19.53^d^	<0.0001
Aortic cross clamp time (minute), (n)	81.12 ± 3.69	91.21 ± 4.23	103.04 ± 8.73	121.73 ± 11.61^b^	0.0023
Preoperative Src (μmol/L)	88.78 ± 2.08	95.44 ± 1.41^a^	86.26 ± 2.98	76.61 ± 3.40^a^	<0.0001
LVEF (%)	58.99 ± 0.74	60.42 ± 0.58	61.37 ± 0.87	60.05 ± 0.81	0.3781
NYHA					0.0158
I (%)	31 (30.39%)	43 (37.39%)	20 (40.82%)	23 (57.50%)	
II (%)	51 (50.00%)	35 (30.43%)	16 (32.65%)	10 (25.00%)	
III (%)	20 (19.61%)	35 (30.43%)	13 (26.53%)	7 (14.28%)	
IV (%)	0	2 (1.74%)	0	0	
ICU LOS (d)	3.43 ± 0.34	3.98 ± 0.28	6.39 ± 1.02^a^	11.00 ± 1.06^d^	<0.0001
Hospitalization	15.37 ± 0.80	17.06 ± 0.82	21.31 ± 1.98^a^	24.08 ± 2.13^c^	0.0001
Ventilator time (h)	19.83 ± 1.63	33.56 ± 3.57^b^	73.24 ± 17.12^d^	97.35 ± 12.95^d^	<0.0001
Dialysis (n)	1	2	6	18	<0.0001
In-hospital death or therapy failure (n)	1	3	6	6	0.0005

*^a^p<0.05; ^b^p<0.01;^c^p<0.001; ^d^p<0.0001. Data are expressed as either Mean ± SDE or as number and percentage. ICU LOS, Length of ICU stay; LVEF, left ventricular ejection fraction.*

### The Most Significantly Correlated Plasma Cytokines With the Postoperative Changes of Serum Creatinine in the CSA-AKI Patients

**FIGURE 3:**
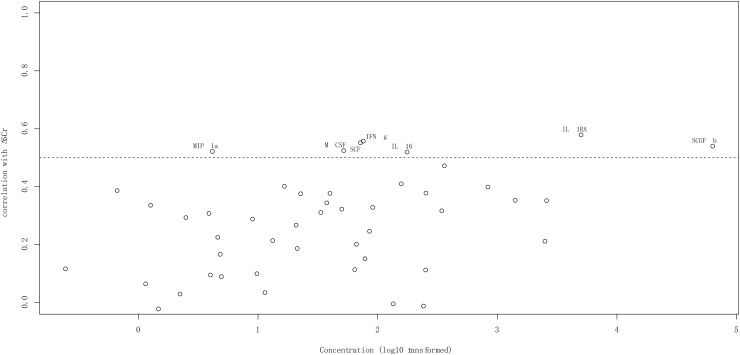
The correlation coefficients of the plasma concentrations of these 48 cytokines with the postoperative ΔSCr.

We assessed the correlation coefficients of the plasma concentrations of these 48 cytokines with the postoperative ΔSCr in all the 306 adult patients with cardiac surgery. We found that 7 out of these 48 cytokines, including IL-1RA, IFN-γ, SCF, SCGF-β, M-CSF, MIP-1α, and IL-16, were the most significantly correlated plasma cytokines with the postoperative ΔSCr, exhibiting the correlation coefficients *r* > 0.5 (Figures 3, 6, Supplementary Figure S3, and Supplementary Table S1). We then performed multivariable logistic regression analysis for clinical factors to assess the prediction of CSA-AKI, and CPB time was independent predictor for AKI. So, we added each biomarker to the clinical factor to determine improvement in the predictive efficiency model. As Table 3 shows, AUC increased when add each factor to CPB time. Especially TGF-β, the value of which could increase to 0.95.

**FIGURE 4:**
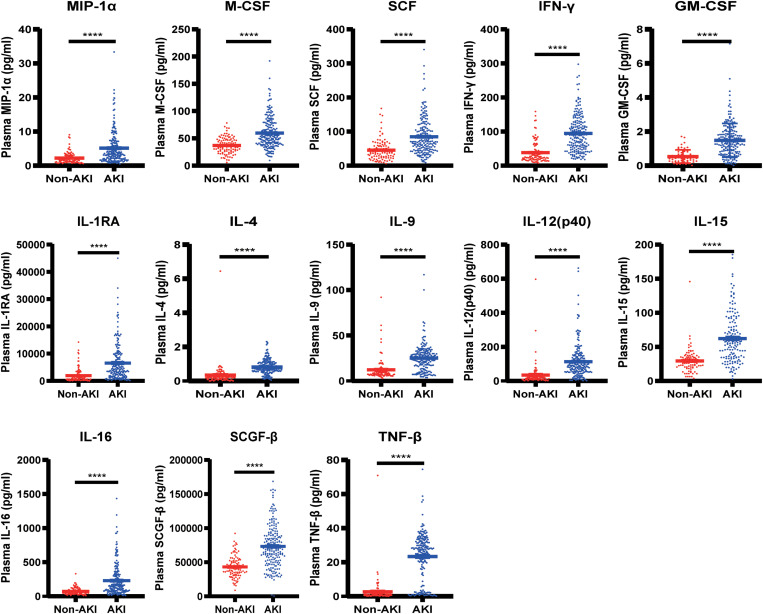
The most significantly changed cytokines between the AKI and non-AKI groups. ****p < 0.00001.

**FIGURE 5:**
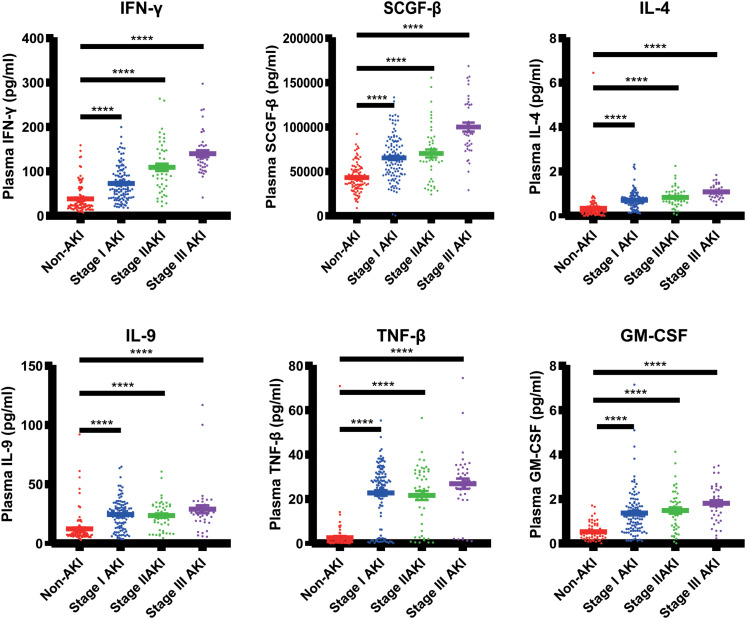
The significantly different cytokines among the non-AKI, Stage I AKI, Stage II AKI, and Stage III AKI groups. ****p < 0.00001.

**FIGURE 6:**
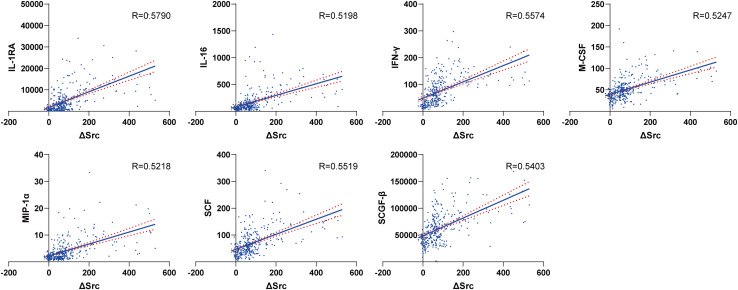
The most significantly correlated plasma cytokines with the postoperative ΔSCr.

**TABLE 3:** The performance of a predictive model after add a factor to clinical model.

	AUC	95% CI
Clinical model	0.7271	(0.6521, 0.8021)
+GM-CSF	0.8063	(0.7419, 0.8708)
+INF-γ	0.8727	(0.8157, 0.9298)
+IL-1RA	0.8305	(0.7683, 0.8928)
+IL-4	0.9048	(0.8569, 0.9526)
+IL-8	0.8595	(0.8028, 0.9161)
+IL-9	0.8042	(0.7983, 0.8687)
+IL-12 (P70)	0.8300	(0.7626, 0.8973)
+IL-12 (P40)	0.9038	(0.8465, 0.9612)
+SCGF-β	0.8766	(0.8249, 0.9284)
+TGF-β	0.9470	(0.9156, 0.9784)

## Discussion

The present prospective cohort study evaluated the diagnostic efficacy of a total of 48 plasma cytokines within the first 24 h after cardiac surgery for the postoperative progression of AKI in 306 adult patients.

AKI occurs in up to 30% of patients who undergo cardiac surgery, but the pathogenesis of CSA-AKI remains unclear (Rosner and Okusa, 2006). Multiple factors, such as hemodynamics, inflammation and nephrotoxin, are thought to be involved and overlapped with each other resulting in renal injury, most likely the tubular necrosis, following an episode of cardiac surgery (Rosner and Okusa, 2006). To date, no pharmacologic interventions or clinical strategies have shown the conclusive efficacy to prevent CSA-AKI. Moreover, the diagnosis of CSA-AKI is still generally based on the criteria of postoperative ΔSCr (Rosner and Okusa, 2006; Thiele et al., 2015; O’Neal et al., 2016; Wang and Bellomo, 2017). Although SCr is considered as the best and golden standard biomarker indicating the changes of renal clearance function, it has been found to poorly correlate with the onset or the initiation of CSA-AKI when the cellular ATP depletion and oxidative injury triggers a proinflammatory response in the kidney while the GFR has not remarkably declined (Ho et al., 2012; Thiele et al., 2015; Bernardi et al., 2016; O’Neal et al., 2016). Therefore, to discover new biomarkers in the initiation phase of CSA-AKI is of great importance for both predictive diagnosis and potential therapeutics.

In a recent prospective cohort study of pediatric CSA-AKI, the urinary and plasma concentrations of a series of cytokines were measured during the early postoperative stage and their correlation coefficients with the progression of CSA-AKI were evaluated, in which the plasma level of IL-8, a potent proinflammatory cytokine, was found to display the best discrimination for the AKI progression after pediatric cardiac surgery (Greenberg et al., 2018). However, neither the cytokines array profiles in the initiation phase after adult cardiac surgery nor their correlation with the development of CSA-AKI have been investigated before.

Therefore, in the present study, we measured the plasma concentrations of 48 cytokines within 24 h after cardiac surgery in a total of 306 adult patients including 204 with and 102 without CSA-AKI. Among the 48 cytokines in the plasma, 20 of them were significantly different in the CSA-AKI patients compared with the non-AKI subjects. In particularly, 13 out of these 20 cytokines including TNF-β, IFN-γ, SCGF-β, IL-15, IL-9, IL-4, M-CSF, GM-CSF, SCF, IL-16, IL-12, IL-1RA, and MIP-1α, displayed tremendous changes with the *P* < 1E^–5^.

Moreover, we compared the levels of 48 cytokines between the patients with different stages of AKI. We found that 10 of the 48 cytokines in the plasma were significantly different among the subjects of non-AKI, Stage I AKI, Stage II AKI, and Stage III AKI. In particularly, 6 out of these 10 cytokines including TNF-β, SCGF-β, IL-9, IFN-γ, GM-CSF, and IL-4, exhibited immense differences with the *P* < 1E^–5^.

To further evaluate the predictive efficacy of these 48 cytokines for the progression of CSA-AKI, we also assessed the correlation coefficients of the plasma concentration of each cytokine with the postoperative ΔSCr from baseline during hospitalization in all the 306 patients included. As shown in Figure 3, among the 48 cytokines, 7 cytokines including IL-1RA, IFN-γ, SCF, SCGF-β, M-CSF, MIP-1α, and IL-16, have the highest correlation coefficient with the *r* > 0.5.

Taken all the results together, we found that IFN-γ and SCGF-β were the most relevant two cytokines that have both the remarkable differences with *P* < 1E^–5^ between AKI and non-AKI patients as well as among subjects with different AKI stages, and the highest correlation coefficients of *r* > 0.5 with the postoperative changes of SCr after cardiac surgery.

SCGF-β (stem cell growth factor β), a newly discovered hematopoietic growth factor exerting its activity at the early stages of hematopoiesis, is rarely investigated in kidney, which indicates that the plasma SCGF-β might be a new biomarker not only for CSA-AKI but also for general AKI (Hiraoka et al., 1997; Mio et al., 1998; Ito et al., 2003). Additionally, IFN-γ (Interferon γ), a well-known pro-inflammatory factor produced mainly by activated T-cells and natural killer cells, has been extensively studied in AKI (Tau and Rothman, 1999; Schroder et al., 2004; Schoenborn and Wilson, 2007). Previous studies have reported that the plasma levels of IFN-γ during the early postoperative stage were either declined or not significantly changed in the kidney transplantation-associated AKI (Karczewski et al., 2009; Snoeijs et al., 2011; De Serres et al., 2012; Xu et al., 2013) and even the pediatric CSA-AKI (Greenberg et al., 2018), which suggests that plasma IFN-γ might be a novel potential biomarker selective for the CSA-AKI in adults.

In conclusion, the plasma levels of IFN-γ and SCGF-β are not only remarkably increased in adult CSA-AKI patients compared with non-AKI patients during the first 24 h after cardiac surgery, but also significantly correlated with the postoperative changes of SCr after cardiac surgery. Thus, IFN-γ and SCGF-β could be novel predictive plasma biomarker, as well as potential therapeutic targets specific for adult CSA-AKI.

There are several limitations to this study. For instance, described by numerous literatures, drugs treating heart failure such as diuretics, ACE inhibitors andAT1R blockers would impact inflammatory pathway. We lack data on drugs used by majority patients prior to cardiac surgery, which would thereafter lead to insufficient diagnostic efficacy of cytokines. Additionally, the circulating cytokines/chemokines are regulated on rapid timescales. Due to sampling difficulties, it is challenging to depict dynamic changes of those designated cytokines at fine resolution.

## Data Availability Statement

The raw data supporting the conclusions of this article will be made available by the authors, without undue reservation.

## Ethics Statement

The studies involving human participants were reviewed and approved by the ethics committee of the Fuwai hospital. The patients/participants provided their written informed consent to participate in this study. Written informed consent was obtained from the individual(s) for the publication of any potentially identifiable images or data included in this article.

## Author Contributions

YL and JS designed the study. ZC and ZH carried out experiments. ZH analyzed the data and made the figures. ZC drafted the manuscript. All authors contributed to the article and approved the submitted version.

## Conflict of Interest

The authors declare that the research was conducted in the absence of any commercial or financial relationships that could be construed as a potential conflict of interest.

## Supplementary Material

Supplementary Table S1 Bonf_P-value between AKI and non-AKI.

Supplementary Table S2 Bonf_P-value between different stage AKI and non-AKI.

Supplementary Table S3 The correlation coefficients of the plasma concentrations of these 48 cytokines with the postoperative ΔSCr.
