# Erratum: Novel potential biomarker of adult cardiac surgery-associated acute kidney injury

**DOI:** 10.3389/fphys.2022.1042372

**Published:** 2022-09-28

**Authors:** Frontiers Production Office

**Affiliations:** Frontiers Media SA, Lausanne, Switzerland

**Keywords:** biomarker, acute kidney injury, cytokines, IFN-γ, SCGF-β

Due to a production error, one of the author names was spelled incorrectly as “Zhengliang Hu”. The correct spelling is “Zhenliang Hu”. The publisher apologizes for this mistake.

The original version of this article has been updated.

