# Supplementary material for: Novel Potential Biomarker of Adult Cardiac Surgery-Associated Acute Kidney Injury
Source: Front Physiol. 2020 Nov 10;11:587204. doi: 10.3389/fphys.2020.587204 (PMC7683426; doi:10.3389/fphys.2020.587204)
Supplement: Supplementary Table S1 — Bonf_P-value between AKI and non-AKI. [file Table_1.docx]

**Table S1. Bonf_P-value between AKI and non-AKI.**

| **Cytokine** | **Bonf_P-value** |
| --- | --- |
| **TNF-b** | **3.66E-31** |
| **IFN-g** | **2.96E-16** |
| **SCGF-b** | **1.33E-14** |
| **IL-15** | **4.18E-11** |
| **IL-9** | **1.14E-10** |
| **IL-4** | **2.04E-10** |
| **M-CSF** | **7.88E-10** |
| **GM-CSF** | **1.05E-09** |
| **SCF** | **6.71E-09** |
| **IL-16** | **1.87E-07** |
| **IL-12** | **2.21E-07** |
| **IL-1RA** | **4.77E-07** |
| **MIP-1a** | **9.00E-06** |
| **CTACK** | **3.11E-05** |
| **SDF-1a** | **5.76E-05** |
| **IL-2Ra** | **0.000245029** |
| **MIF** | **0.002173241** |
| **TNF-a** | **0.004307462** |
| **VEGF-A** | **0.031875655** |
| **MIP-1b** | **0.032105029** |
| **IL-5** | **0.051064362** |
| **IL-10** | **0.066684785** |
| **HGF** | **0.082338645** |
| **GRO-a** | **0.353348932** |
| **FGF basic** | **0.608198148** |
| **IL-2** | **0.82009823** |
| **Eotaxin** | **1** |
| **G-CSF** | **1** |
| **IFN-a2** | **1** |
| **IL-1a** | **1** |
| **IL-1b** | **1** |
| **IL-3** | **1** |
| **IL-6** | **1** |
| **IL-7** | **1** |
| **IL-8** | **1** |
| **IL-12** | **1** |
| **IL-13** | **1** |
| **IL-17A** | **1** |
| **IL-18** | **1** |
| **IP-10** | **1** |
| **LIF** | **1** |
| **MCP-1** | **1** |
| **MCP-3** | **1** |
| **MIG** | **1** |
| **b-NGF** | **1** |
| **PDGF-BB** | **1** |
| **RANTES** | **1** |
| **TRAIL** | **1** |

**Table S2. Bonf_P-value between different stage AKI and non-AKI.**

| **Cytokine** | **Bonf_P-value** |
| --- | --- |
| **TNF-b** | **1.03E-25** |
| **SCGF-b** | **1.81E-08** |
| **IL-9** | **3.06E-08** |
| **IFN-g** | **2.10E-07** |
| **GM-CSF** | **9.17E-07** |
| **IL-4** | **6.55E-06** |
| **IL-15** | **1.11E-05** |
| **M-CSF** | **0.000324968** |
| **IL-12** | **0.001058627** |
| **SCF** | **0.003530286** |
| **IL-16** | **0.181199289** |
| **SDF-1a** | **0.193682781** |
| **MIP-1a** | **0.421743714** |
| **IL-1RA** | **0.461577942** |
| **CTACK** | **0.689964359** |
| **Eotaxin** | **1** |
| **FGF basic** | **1** |
| **G-CSF** | **1** |
| **GRO-a** | **1** |
| **HGF** | **1** |
| **IFN-a2** | **1** |
| **IL-1a** | **1** |
| **IL-1b** | **1** |
| **IL-2** | **1** |
| **IL-2Ra** | **1** |
| **IL-3** | **1** |
| **IL-5** | **1** |
| **IL-6** | **1** |
| **IL-7** | **1** |
| **IL-8** | **1** |
| **IL-10** | **1** |
| **IL-12** | **1** |
| **IL-13** | **1** |
| **IL-17A** | **1** |
| **IL-18** | **1** |
| **IP-10** | **1** |
| **LIF** | **1** |
| **MCP-1** | **1** |
| **MCP-3** | **1** |
| **MIF** | **1** |
| **MIG** | **1** |
| **MIP-1b** | **1** |
| **b-NGF** | **1** |
| **PDGF-BB** | **1** |
| **RANTES** | **1** |
| **TNF-a** | **1** |
| **TRAIL** | **1** |
| **VEGF-A** | **1** |

**Table S3. The correlation coefficients of the plasma concentrations of these 48 cytokines with the postoperative ΔSCr**

| **Cytokine** | **r** |
| --- | --- |
| **IL-1RA** | **0.579043581** |
| **IFN-g** | **0.557361837** |
| **SCF** | **0.551857576** |
| **SCGF-b** | **0.540306424** |
| **M-CSF** | **0.524661112** |
| **MIP-1a** | **0.521757588** |
| **IL-16** | **0.519816439** |
| **CTACK** | **0.472289016** |
| **IL-2Ra** | **0.410178628** |
| **TNF-b** | **0.401602016** |
| **MIF** | **0.39894452** |
| **IL-4** | **0.386755334** |
| **SDF-1a** | **0.378135614** |
| **MIP-1b** | **0.377253996** |
| **TNF-a** | **0.375789127** |
| **MIG** | **0.352968778** |
| **IP-10** | **0.351950106** |
| **IL-10** | **0.34436722** |
| **GM-CSF** | **0.335977201** |
| **IL-12** | **0.328787207** |
| **IL-15** | **0.322491039** |
| **IL-6** | **0.316982762** |
| **VEGF-A** | **0.311046896** |
| **IL-2** | **0.308008808** |
| **MCP-3** | **0.293520726** |
| **IL-5** | **0.288203936** |
| **IL-9** | **0.267388902** |
| **MCP-1** | **0.246442117** |
| **IFN-a2** | **0.225488006** |
| **FGF basic** | **0.214110353** |
| **HGF** | **0.211902442** |
| **Eotaxin** | **0.201306085** |
| **TRAIL** | **0.186860807** |
| **IL-17A** | **0.166709386** |
| **GRO-a** | **0.151413501** |
| **IL-3** | **0.116692801** |
| **IL-8** | **0.113975714** |
| **G-CSF** | **0.112512733** |
| **LIF** | **0.09967821** |
| **IL-7** | **0.095368862** |
| **IL-12** | **0.089623203** |
| **IL-1b** | **0.064965062** |
| **IL-1a** | **0.034452061** |
| **IL-13** | **0.029649861** |
| **IL-18** | **-0.004323799** |
| **PDGF-BB** | **-0.011815484** |
| **b-NGF** | **-0.02179121** |
| **RANTES** | **-0.084435072** |
