# Supplementary figures and images for: Novel Potential Biomarker of Adult Cardiac Surgery-Associated Acute Kidney Injury
Source: Front Physiol. 2020 Nov 10;11:587204. doi: 10.3389/fphys.2020.587204 (PMC7683426; doi:10.3389/fphys.2020.587204)

Figure S1

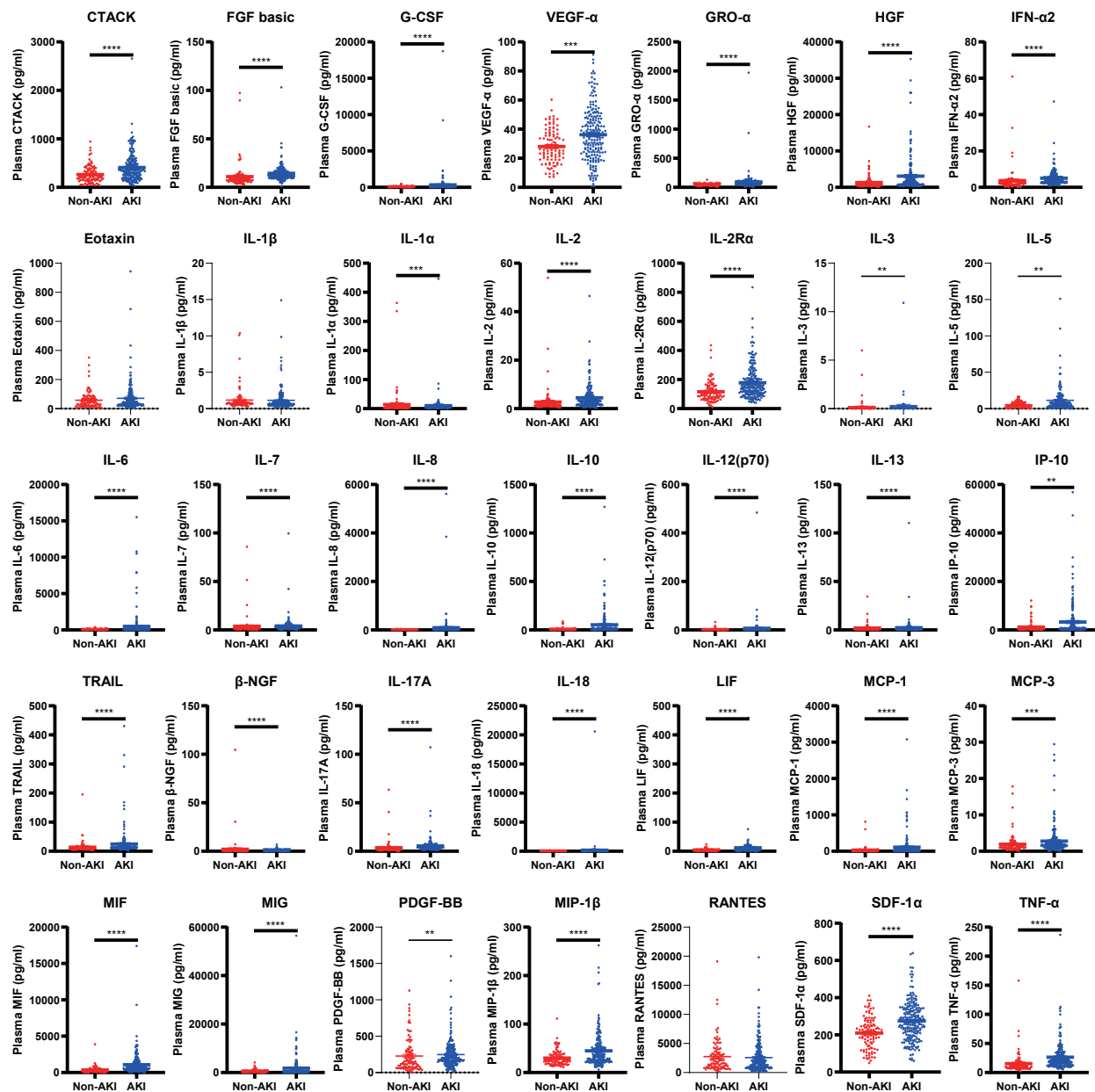

Fig S1. The plasma concentrations of left 35 cytokines in CSA-AKI and non-AKI group.

\* p < 0.05, \*\* p < 0.01, \*\*\* p < 0.001, \*\*\*\* p < 0.0001.

Supplement: Supplementary file 2 [file Image_1.pdf]
