# Supplementary material for: Novel Potential Biomarker of Adult Cardiac Surgery-Associated Acute Kidney Injury
Source: Front Physiol. 2020 Nov 10;11:587204. doi: 10.3389/fphys.2020.587204 (PMC7683426; doi:10.3389/fphys.2020.587204)
Supplement: Supplementary file 3 [file Image_2.pdf]

Figure S2

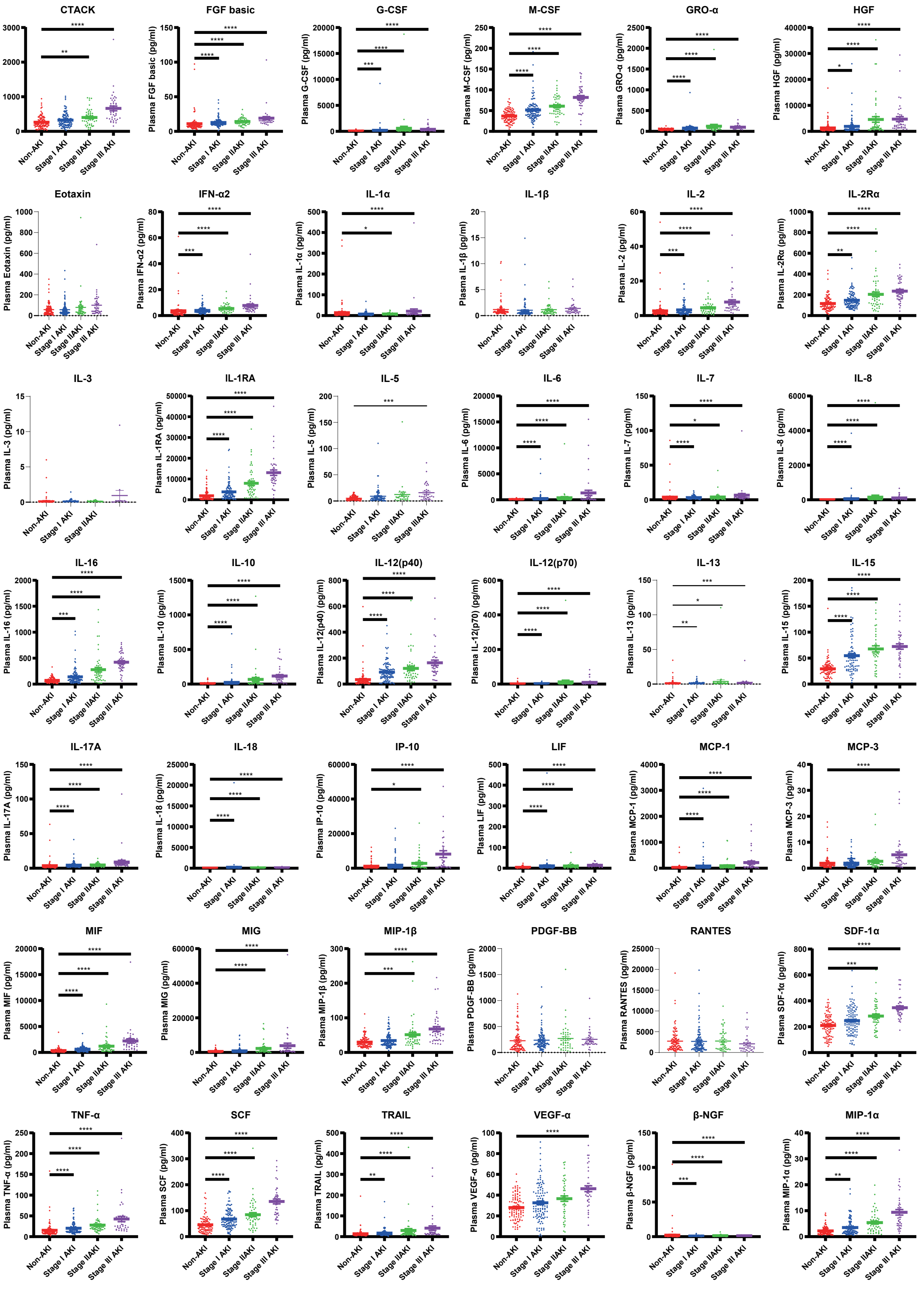

Fig S2. The plasma concentrations of left 42 cytokines in different AKI stages and non-AKI group. * p<0. 05, ** p<0. 01, *** p<0. 001, **** p<0. 0001.
