# Supplementary material for: Novel Potential Biomarker of Adult Cardiac Surgery-Associated Acute Kidney Injury
Source: Front Physiol. 2020 Nov 10;11:587204. doi: 10.3389/fphys.2020.587204 (PMC7683426; doi:10.3389/fphys.2020.587204)
Supplement: Supplementary file 4 [file Image_3.pdf]

Figure S3

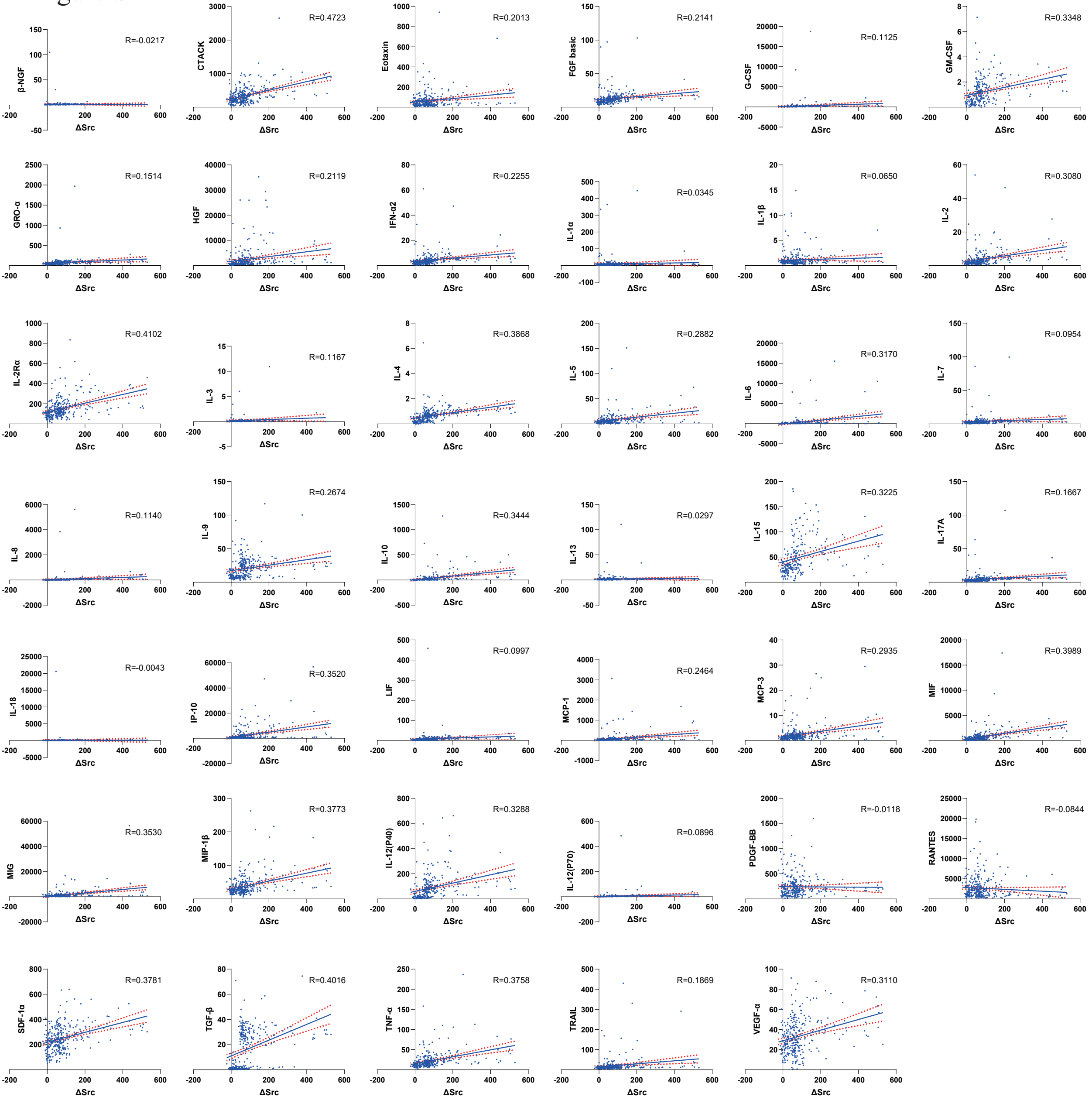

Fig S3. The correlation coefficients of the plasma concentrations of left 41 cytokines with the postoperative ΔSCr.
